# On suicidal ideation: the need for inductive methodologies to advance the field

**DOI:** 10.47626/2237-6089-2023-0655

**Published:** 2023-10-31

**Authors:** Lauro Estivalete Marchionatti, Pedro Vieira da Silva Magalhães

**Affiliations:** 1 Programa de Pós-Graduação em Psiquiatria e Ciências do Comportamento Faculdade de Medicina Universidade Federal do Rio Grande do Sul Porto Alegre RS Brazil Programa de Pós-Graduação em Psiquiatria e Ciências do Comportamento, Faculdade de Medicina, Universidade Federal do Rio Grande do Sul, Porto Alegre, RS, Brazil.; 2 Centro de Pesquisa Clínica Hospital de Clínicas de Porto Alegre Porto Alegre RS Brazil Centro de Pesquisa Clínica, Hospital de Clínicas de Porto Alegre, Porto Alegre, RS, Brazil.

**Keywords:** Suicide, suicidal ideation, qualitative research

## Abstract

Recent scholarly investigation of suicidal ideation has been largely based on identifying associated factors and using ideation-to-action theories to explain its occurrence. However, this approach may not be sufficient, as many aspects of suicidal ideation fall beyond the reach of such conceptualizations. The overemphasis on explaining rather than understanding this phenomenon is a significant factor in this insufficiency. As such, it is argued that qualitative methods that use data to derive theories could offer a more nuanced understanding of suicidal ideation. By adopting bottom-up approaches, researchers can explore how individuals experience and understand suicidal ideation and how it relates to their lives and experiences. Furthermore, use of qualitative research methods could aid in development of more accurate and inclusive definitions that are more firmly grounded in data.

Suicidal ideation is a worldwide burden that is part of the mental health crisis^1^ and current theories suggest that it is a crucial node in the path leading to suicide.^2^ Nevertheless, the term can be used in significantly different ways. For some authors, suicidal ideation includes passive thoughts of death,^3-5^ while others define it as ideas of intentionally taking one’s own life.^6,7^ There are similar controversies relating to suicidal intention, with some considering it a separate step,^3^ while others classify plans and mental rehearsals within the scope of suicidal ideation,^6^ pose intention as a specifier of ideation,^8^ or state that it is not currently useful to distinguish between these constructs.^9^

Such divergence could be attributable to insufficient exploration of the subject. When scholars and clinicians consider suicidal ideation mainly through a diagnostic lens and attribute it to depressive symptomatology,^10,11^ phenomenological aspects such as triggers or meanings can be neglected. This is analogous in research, as most studies circumscribe ideation to a marker of suicide risk or merely inspect its associated factors.^12^ Many publications also deal with suicidal phenomena as manifestations of varying intensity along the same continuum,^13^ disregarding the possibility that they could be categorically distinct.^14^ As such, there are questions about whether many of the risk factors described for suicide actually constitute suicidal ideation^2^ and indeed studies have not been able to predict which individuals with ideation will actually attempt suicide.^15-17^

Notwithstanding such difficulties, conceptual models of suicide do distinguish ideation as a separate step at the beginning of a process that may lead to enaction.^2^ This was first proposed in the Interpersonal Theory of Suicide,^18^ which claims that suicidal ideation results from “thwarted belongingness” and “perceived burdensomeness,” progressing to an attempt in the presence of “acquired capacity.” Subsequent theories emerged within the ideation-to-action framework, usually explaining the genesis and progression of suicidal ideation by a combination of factors. The Integrated Motivational-Volitional Model proposes that suicidal ideation and intention result from defeat, humiliation, and entrapment,^9^ whilst the Three-Step Theory (3ST) attributes suicidal ideation to pain (usually psychological) and hopelessness, with intensity moderated by sense of belonging.^19^

Regardless of the accuracy of proposals of a determined set of factors as giving rise to suicidal ideation, these dominant models may end up oversimplifying the phenomenon. They fail to capture aspects of suicidal ideation that have been raised in other work. For instance, the developer of the Dialectical-Behavior Therapy, Linehan,^20^ proposes that “suicide ideation, suicide planning, and imagining dying from suicide, when accompanied with a belief that pain will end with death, can bring an intense sense of relief,” and that, “planning suicide, imagining suicide, and engaging in a self-injurious act (and its aftereffects if it becomes public) can reduce painful emotions by providing a compelling distraction,” thereby proposing a function for suicidal ideation, which is a feature that is not mentioned by current models, which limit their scope to its emergence and progression. Indeed, a few quantitative studies have endorsed suicidal cognition as a source of relief.^21-23^ Another instance of phenomenological aspects of suicidal ideation that are not contemplated in the dominant models of suicide is the presence of detailed mental imagery of suicide that may be more prominent than verbal thoughts.^22,23^

The reason for the insufficiency of these models may lie in over-reliance on explaining a phenomenon that ought first to be understood. Indeed, the dominant paradigm for such models is hypothetical-deductive, in which researchers first formulate factors and then look for empirical corroboration in data,^2^ usually using scales to measure the levels of the proposed components among suicidal ideators.^24^ Similarly, the underperformance of predictive models of suicidal behavior has been attributed to applying statistical modeling to a limited set of variables of a phenomenon that still warrants greater understanding.^25,26^ Indeed, suicidology authors have called attention to the need to step back, avoiding reductionism and broadening the range of methodologies used to research suicidal ideation.^12,27,28^

An alternative road would involve inspecting data and then deriving a theory, in a bottom-up process that inquires ideators and uses their reports to understand, characterize, and conceptualize suicidal ideation.^29,30^ Such an inductive approach is usually associated with qualitative research, which is an approach recommended for deepening comprehension of phenomena that are insufficiently explored,^31^ traditionally locating the lived experience of individuals as the point of departure for knowledge building.^27,32^ Indeed, extant inductive inquiries investigating suicidal ideation have proved quite fruitful, presenting novel findings that are beyond the reach of current theories in the field. For instance, Denneson et al.^33^ interviewed 50 people in the United States and arrived at several novel insights on the nature of suicidal ideation. They revealed that suicidal ideation is a chronic symptom, usually perceived to be ever present over the course of years and considered to be beyond the control of the individuals experiencing it and often unpredictable. Similar research was conducted with older Taiwanese adults, endorsing the observation that suicidal ideation can linger on over many years and also calling attention to religion as an important factor in averting attempts.^34^ Another qualitative study investigated how psychotherapy helped Canadian adolescents by conducting content analysis of interviews, framing suicidal ideation as a way of coping with distress in the context of an undeveloped repertoire of other strategies.^35^ Similarly, work on suicidal ideation was conducted with LGBTQIA+ men in New York who had been recently diagnosed with human immunodeficiency virus (HIV).^36^ This study concluded that suicidality provoked a process of coping with the diagnosis by enhancing one’s sense of control over life, which ultimately led to a positive reattribution of meanings associated with HIV and acceptance.

There are many possibilities for advancing comprehension of suicidal ideation to be expected from employment of bottom-up methodologies. Previous research has revealed that inductive methods can yield novel insights into the constitution of suicidal ideation as a symptom. In this direction, this venture could achieve a deeper understanding of suicidal ideation’s manifestations, course, triggers, function, and coping strategies, also informing treatment. This approach is reminiscent of phenomenology’s emphasis on observation of subjective experience, which has yielded rich descriptions of psychopathology that significantly contribute to our knowledge of mental health disorders such as schizophrenia.^37,38^ A more comprehensive understanding of suicidal ideation could also arrive at a definition that is more firmly grounded in data, since the field currently struggles with top-down (and often incompatible) definitions of the term that strictly define boundaries. Moreover, such an exploration could reveal important aspects of the relation of suicidal ideation to suicide, which would better situate the phenomenon within current theoretical models. Finally, critical suicidology has long argued against reducing the highly contextual phenomenon of suicidal ideation to a static panel of risk factors.^28,39^ Methodologies that account for the lived experience can embrace this complexity and shed new light on the ways in which contextual factors shape and interplay with the symptom.
